# Pulmonary hypertension among patients undergoing hemodialysis

**DOI:** 10.15171/jrip.2017.24

**Published:** 2016-12-04

**Authors:** Fatemeh Hayati, Seyed Seifollah Beladi Mousavi, Seyed Majid Mousavi Movahed, Maryam Mofrad Bushehri

**Affiliations:** ^1^Chronic Renal Failure Research Center, Ahvaz Jundishapur University of Medical Sciences, Ahvaz, Iran; ^2^Department of Internal Medicine, Tehran University of Medical Sciences, Tehran, Iran

**Keywords:** Hemodialysis, Pulmonary hypertension, End-stage renal disease

## Abstract

**Introduction:** The epidemiology of pulmonary hypertension (PHT) among long-term hemodialysis patients has been described in relatively small studies in Iran.

**Objectives:** The purpose of this study was to evaluate the prevalence of PHT and its relationship among end-stage renal disease (ESRD) patients undergoing long-term hemodialysis (HD).

**Patients and Methods:** In a cross-sectional study, patients with ESRD treated with HD for at least 3 months in the Imam hospital enrolled for the study. PHT was defined as an estimated systolic pulmonary artery pressure (PAP) equal to or higher than 25 mm Hg using echocardiograms performed by cardiologist.

**Results:** A total of 69 HD patients were included in the investigation. The mean of age of our patients was 52.6±15.3 years. The mean duration of HD was 39±36 months. The mean ejection fraction was 45±7%. The prevalence of PHT was 62.3%. These patients were more likely to have lower ejection fraction. The PHT was more common among female HD patients. We did not find any association between PHT and cause of ESRD, duration of HD, anemia and serum calcium, phosphor and parathyroid hormone levels.

**Conclusion:** Our findings show that PHT is a common problem among ESRD patients undergoing maintenance HD and it is strongly associated with heart failure. It is necessary to screen this disorder among these patients.

**Implication for health policy/practice/research/medical education:**

It seems that PHT is a common disorder in ESRD patients and it is also associated with poor outcomes and reduced survival among these patients. Therefore prevention, early diagnosis and treatment of PHT are attractive target to improve survival of these patients. However, the true prevalence of PHT among ESRD and HD patients is unknown and its epidemiology has been described only in relatively small studies. In a study on 69 HD patients, we found that the PHT is a common disorder and more than 60% of ESRD patients undergoing maintenance HD have PHT. It is more common among female patients and ESRD patients with PHT have lower ejection fraction in comparison with other HD patients.

## Introduction

Pulmonary hypertension (PHT) is characterized by a mean pulmonary artery pressure (PAP) equal to or higher than 25 mm Hg (1-3). Most of the patients with PHT initially present with fatigue, decreased exercise tolerance and exertional dyspnea and if untreated it can be a progressive, severe and fatal disease (2-4). According to etiology and mechanism, World Health Organization (WHO) have classified PHT into the five groups including idiopathic pulmonary arterial hypertension (group 1), PHT due to left heart disease (group 2), PHT due to chronic lung disease and/or hypoxemia (group 3), chronic thromboembolic PHT (group 4) and PHT due to unclear multifactorial mechanisms (group 5) (1-4).

PHT with unclear multifactorial mechanisms, group 5, include patients with PHT caused by chronic kidney disease and other disorders like chronic hemolytic anemia, sickle cell disease, myeloproliferative disorders, sarcoidosis and metabolic disorders like glycogen storage disease (2-4).

The true prevalence of PHT among chronic kidney disease patients is unknown and its epidemiology has been described only in relatively small studies. However it seems that PHT is not only a common disorder among these patients but also it is associated with poor outcomes and reduced survival (1-4). Therefore prevention, early diagnosis and treatment of PHT are attractive target to improve survival of these patients.

## Objectives

The purpose of this study was to evaluate the prevalence of PHT and its relationship among end- stage renal disease (ESRD) patients undergoing long-term hemodialysis (HD).

## Patients and Methods

### Study design

The study was a cross-sectional, from May 2014 to March 2015, which approved by the Ethics Com­mittee of the Chronic Renal Failure Research Center of Ahvaz Jundishapur University of Medical Sciences Ahvaz, Iran.

A standardized questionnaire was used to collect demographic data and data regarding the kidney disease and HD including etiology of ESRD, date of onset of renal replacement therapy (RRT) and HD access. Laboratory data including hemoglobin, calcium, phosphor and parathyroid hormone level was also collected.

### Patients

ESRD patients ≥18 years old had been on maintenance HD for >2 months in Ahvaz Imam-Khomaini hospital in the province of Khuzestan. The ESRD was defined as irreversible and advance loss of kidney function due to any etiology requiring long term RRT with HD (5-9).

HD patients with the following characteristics, patients who had mitral or aortic stenosis, female patients who were pregnant, active or chronic infections and patients who had severe chronic obstructive pulmonary disease (COPD) were excluded from the study (1-3).

### Hemodialysis method

In our center, HD was performed for ESRD patients one, two or three times a week, for about 4 hours for each session using Fresenius machines. The most of the patients were treated with synthetic (polysulfone) dialyzer membranes and bicarbonate-based dialysis solution at a delivered bicarbonate concentration of 35-40 mEq/L. The blood flow rate and the dialysate flow rate were 200-400 mL/min and 500 mL/min respectively.

### Measurements

#### Echocardiograms

Two-dimensional (2D) and guided M-mode echocardiogram were performed by cardiologist with a digital cardiac ultrasound machine (Biosound Esaote, Indianapolis, USA) as a noninvasive method. PHT was defined as a mean PAP equal to or higher than 25 mm Hg.

#### Laboratory assay

Blood sampling of our patients for assay of hemoglobin, calcium, phosphor and parathyroid hormone level was assessed immediately before the dialysis session by staffs of HD and the serum was separated without delay. Immunoradiometric assays (second generation parathyroid hormone assays) was used for check of serum intact parathyroid hormone (iPTH) levels.

### Ethical issues

1) The research followed the tenets of the Declaration of Helsinki; 2) informed consent was obtained, and they were free to leave the study at any time and 3) the research was approved by the ethical committee of the Chronic Renal Failure Research Center of Ahvaz Jundishapur University of Medical Sciences Ahvaz, Iran.

### Statistical analysis

For data analysis, we used the SPSS version 15 software. Descriptive statistics for demographic and laboratory variables including hemoglobin, calcium, phosphor and parathyroid hormone level, related to the prevalence of PHT were calculated.

To test the association of quantitative and qualitative variables with PHT, chi-square tests or Fisher’s exact and *t* test were performed. Statistical significance was considered at the *P* value of <0.05 in all statistical analysis.

## Results

Sixty-nine ESRD patients undergoing maintenance HD (33 females and 36 males) with mean age of 52.6 ± 15.3 years were enrolled to the study. The range of PAP was 18 mm Hg to 65 mm Hg.

Regarding a mean PAP equal to or higher than 25 mm Hg as a PHT, 43 patients (62.31%) had PHT. Mean PAP was normal in 24 patients (34.78%) and lower than normal in 2 patients (2.89%).

In comparison with patients with normal PAP, PHT was more common among female patients (*P* = 0.021; Table 1). Mean PAP among female and male patients were 34± 11 mm Hg and 28± 7 mm Hg respectively which higher values in female patients.

**Table 1:** Association of all of variables with PHT

**Variables**	**Statistical methods** ^a^	***P***
Age	Pearson’s test	0.700
Hypertension	*t* test	0.110
Dialysis access	ANOVA Test	0.186
Gender	*t* test	0.02
Duration of dialysis	Pearson’s test	0.270
Proportion of dialysis per week	Spearman’s test	0.273
Hemoglobin(g/dL)	Pearson’s test	0.342
Serum Ca (mg/dL)	Pearson’s test	0.146
Serum phosphorus (mg/dL)	Pearson’s test	0.681
Ejection fraction (%)	Pearson’s test	0.001
Parathyroid hormone level (ρg/mL)	Pearson’s test	0.138
Diabetes	*t* test	0.708

Abbreviation: PHT, pulmonary hypertension. ^a^ To investigate the relationship of these variables with pulmonary artery pressure (PAP).

**Figure 1:**
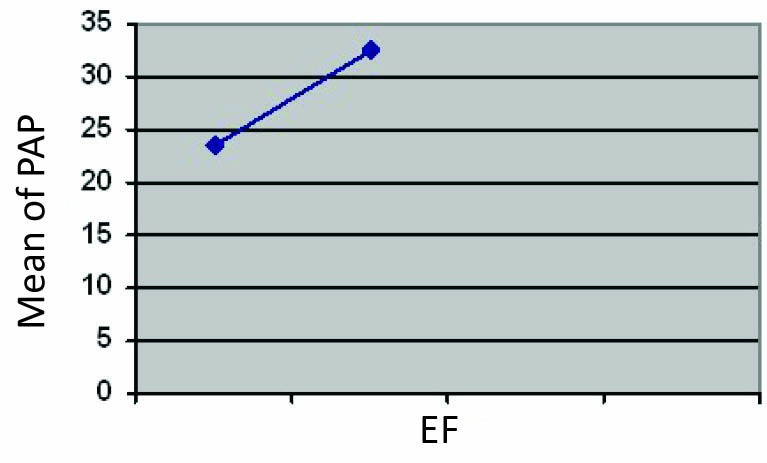
Association of ejection fraction with pulmonary hypertension.

The range of ejection fraction among our patients was 20% to 55% and patients with PHT had lower ejection fraction in comparison with other HD patients (P = 0.001; Figure 1; Table 1).

The cause of ESRD was diabetes in 26 patients (37.68%) and other (43 patients) (62.31%) did not have diabetic nephropathy. In addition 53 patients (76.81%) had hypertension and 16 patients (23.18%) were not hypertensive. There was no association between presence of diabetes (*P* = 0.70) and or hypertension (*P* = 0.11) with the presence of PHP (Table 1).

**Figure 2:**
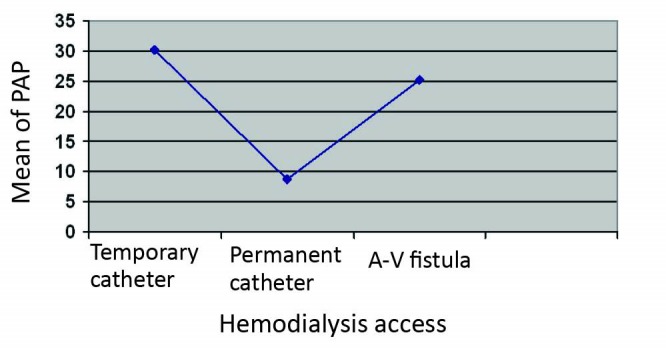
Association of hemodialysis access with pulmonary hypertension.

The HD access in our patients was A-V fistula in 27 patients (39.13%), temporary catheter in 39 patients (56.52%) and permanent catheter in 3 patients (4.37%). There was no association between HD access with PHP (*P* = 0.18; Figure 2).

According to serum parathyroid hormone level, we divided our patients in three groups, patients with normal serum iPTH level (150-300 pg/mL), patients with high serum parathyroid hormone level (higher than 300 pg/mL) and patients with low serum parathyroid hormone level (lower than 150 pg/mL) and then evaluated the relationship of these groups with PHT with use of Pearson’s test. We did not find any association between these groups with PHT (*P* = 0.13; Table 1).

In addition, there was not any association between age (*P* = 0.70), serum Ca (*P* = 0.14), serum phosphorus (*P* = 0.68), levels of hemoglobin (*P* = 0.34), duration of dialysis (P = 0.27) and proportion of dialysis per week (*P* = 0.27) with the presence of PHT (Table 1).

## Discussion

Although maintenance dialysis improves the survival of patients with ESRD, however the outcome of these patients is catastrophic and is much worse than the general population (10-13). For example, according to the report of US Renal Data System, the survival of these patients are approximately 8 and 4.5 years for individuals aged 40 to 44 and 60 to 64 years respectively which are much lower than general population. The major factor of poor outcome of ESRD patients is more prevalence and severity of cardiovascular diseases including PHT among these patients (11-13).

There are various investigations which have considered the relationship of PHT with overall survival of ESRD patients. The results of these studies have showed that PHT has association with increased all-cause mortality among these patients. It has also suggested that its association with poor survival extends to a period beyond dialysis and also after kidney transplantation (14-16).

Our study showed, PHT is a common disorder among ESRD patients. According to the results of the present study, more than 60% of ESRD patients undergoing maintenance HD have PHT and it is more common among female patients. In addition, we showed that ESRD patients with PHT have lower ejection fraction in comparison with other HD patients.

The reported prevalence of PHT in the other studies among ESRD patients on maintenance HD is also high similar to the results of our study (17-22). For example, Beladi Mousavi et al screened 62 ESRD patients on long-term HD for PHT by Doppler echocardiography. In this study, PHT was defined as PAP higher than 35 mm Hg which was higher than threshold of our study. They showed that approximately 50% of ESRD patients on long-term HD have PHT (22).

The pathogenesis of PHT among ESRD patients is unknown, however it has proposed that metabolic and endocrine abnormalities which result from ESRD including calcium × phosphorus metabolism abnormalities, increased stiffness of the pulmonary capillaries due to hyperparathyroidism and pulmonary vascular calcification may contribute to PHT among these patients (21-23).

The results of Amin et al study supported this hypothesis and showed that parathyroid hormone could cause calcification of the pulmonary artery and subsequently PHT in dogs with experimental chronic kidney failure. However, the results of our study and also the results of some other studies including Amin et al study did not show any association between hyperparathyroidism and PHT among these patients (24).

It is also proposed that calcium × phosphorus metabolism abnormalities which are common in ESRD patients may contribute to calcification of the pulmonary artery and subsequently PHT among these patients. However according to the results of other studies including the results of our investigation, levels of calcium, phosphorus and calcium-phosphorus product do not differ significantly between the ESRD patients with and without PHT.

It has also been suggested that arteriovenous (AV) access-mediated increases in cardiac output contributes to the development of PHT among these patients (25-27). The results of Dolmatch et al supported the role of AV fistula and they showed return of elevated PAP and cardiac output towards normal following the ligation of a functioning AV fistula (26). Although, the result of these studies are interesting, however this hypothesis has also not supported by other studies. Therefore, it is possible that other mechanisms and or all of these mechanism may contribute to PHT among ESRD patients.

In fact, PHT is associated with poor outcomes, reduced survival and increased all-cause mortality among ESRD patients. It has also suggested that its association with poor survival extends to a period beyond dialysis and also after kidney transplantation. Therefore prevention, early diagnosis and treatment of PHT are attractive target to improve survival of these patients. However, true prevalence of PHT and its relationship among chronic kidney disease patients has been described only in relatively small studies in Iran.

## Conclusion

The results of our study showed that the PHT is a common disorder and more than 60% of ESRD patients undergoing maintenance HD have PHT. It is more common in female patients under HD.

We did not find any association between cause of ESRD, type of HD access, duration of HD, the number of HD per week, serum parathyroid hormone, serum Ca, serum phosphorus and hemoglobin levels with PHT.

## Limitations of this study

Although the results of our study are interesting, however our study is limited by the short duration and small proportion of patients enrolled to this study. Therefore a multicenter study with long duration and larger proportion of patients is necessary to better evaluation of PHT among ESRD patients.

## Acknowledgments

The authors wish to thank the dean of Chronic Renal Failure Research Center of Ahvaz Jundishapur University of Medical Sciences for his help in financial support and data collection.

## Authors’ contribution

All authors of the manuscript participated equally in conducting and preparing of the study.

## Conflicts of interest

The authors declared no competing interests.

## Ethical considerations

Ethical issues (including plagiarism, data fabrication, double publication) have been completely observed by the authors.

## Funding/Support

This study was extracted from M.D thesis of Maryam Mofrad-Bushehri (Thesis number # E-835). The study was funded by a research grant from the Chronic Renal Failure Research Center of Ahvaz Jundishapur University of Medical Sciences.
